# 

**Published:** 1876-10

**Authors:** 


					﻿The following works have been received, and will be noticed
in a future issue of the Journal.
An Introduction to Pathology and Morbid Anatomy. By T. Henry
Green, M.D., London; Fellow of the Eoyal College of Phystcians,
London; Physician to Charing Cross Hospital Medical School; As-
sistant Physician to the Hospital for Consumption and Diseases of
the Chest, Brompton. Second American, from the third revised and
enlarged English Edition. Philadelphia: Henry C. Lea. 1876.
A Treatise on the Science and Practice of Midwtfery. By. W. S.
Playfair, M.D., F.E.C.P., Professor of Obstetric Medicine in King’s
College; Physician for the Diseases of Women and Children to King’s
College Hospital; Examiner in Midwifery to the University of London;
State Examiner in Midwifery to the Eoyal College of Physicans; and
Vice-President of the Obstetrical Society of London. With two
Plates and one 'hundred and sixty-six Illustrations on Wood. Phila-
delphia: Henry C." Lea. 1876.
A Treatise on the Science and Practice of Mid'wtfery. By. W. S.
Playfair, M.D., F.R.C.P., Professor of Obstetric Medicine in King’s
College; Physician for the Diseases of Women and Children to King’s
College Hospital; Examiner in Midwifery to the University of London;
State Examiner in Midwifery to the Royal College of Physicans; and
Vice-President of the Obstetrical Society of London. With two
Plates and one 'hundred and sixty-six Illustrations on Wood. Phila-
delphia: Henry C." Lea. 1876.
Lectures on Feveb, Deliveeed in the Theatee of Meath Hospital and
County of Dublin Infirmary. By William Stokes, M.D., D.C.S.,
Oxon., F.R.; Begins Professor of Physic in the University of Dublin;
Physician to the Queen in Ireland. Edited by John William Moore,
M.D., F.K.Q.C.P., Physician to the Cook Street Fever Hospital; Ex-
Scholar and Diplomat in State Medicine of Trinity College, Dublin.
Philadelphia: Henry C. Lea. 1876.
The Pathology and Tbeatment of Childbed: A Treatise fob Physi-
cians AND Students. By Dr. F. Winckel, Professor and Director of
the Gynaecological Clinic at the University of Bostock. From the
Second German Edition, with many additional notes by the Author.
Translated by James E. Chadwick, M.D., Clinical Lecturer on Dis-
eases of Women, Harvard University. Philadelphia: Henry C.
Lea. 1876.

				

